# Succesful treatment of food allergy with Nambudripad's Allergy Elimination Techniques (NAET) in a 3-year old: A case report

**DOI:** 10.1186/1757-1626-1-166

**Published:** 2008-09-19

**Authors:** Caroline B Terwee

**Affiliations:** 1 VU University Medical Center, Department of Epidemiology and Biostatistics, EMGO Institute, Amsterdam, The Netherlands

## Abstract

Food allergy may constitute a major burden to children and their families. A 3-year-old girl was intolerant to milk, sugar, egg white, pork meat, and other foods, causing eczema and dyspnoe. She was treated with Nambudripad's Allergy Elimnation Technique (NAET), a combination of kinesiology and acupressure. After 7 treatment sessions (within 4 weeks) she was free of symptoms. After three years, she can still eat everything without symptoms. This case report highlights the possible benefit of NAET for children with food allergy. Randomized clinical trials should be encouraged to study the effectiveness of NAET in treating food allergy.

## Background

Food allergy occurs in 4–6% of young children en is a major cause of life-threatening hypersensitivity reactions [1]. Accidental ingestions of food allergens are common because of the ubiquitous presence of certain foods, such as milk, sugar, peanut, and egg, and compounded by poor labelling practices and cross-contamination during processing [2]. Food allergy may constitute a major burden to children and their families. Children may suffer from their restricted diet, being unable to eat certain foods (often attractive foods such as sweets, cookies, and ice-cream), to accept treats at parties, or to eat at a friends' house. Parents may suffer from fear of food-induced anaphylaxis and feelings of pity or guilt. Furthermore, diets are often costly and time-consuming. In cases of multiple food allergies, restricted diets may result in unbalanced nutrition causing a variety of health problems. Considering the burden and increasing prevalence of food allergies, improved diagnostic tests and treatments are obviously needed.

## Case presentation

A 3-year-old Caucasian girl suffered from food allergy since she had a respiratory syncytial virus infection at the age of 6 months. She was intolerant to milk, sugar, egg white, pork meat, and a few other foods. This was established by elimination-challenge tests. Ingestions of these foods caused eczema on the upper back after 48 hours, followed by snivelling, coughing and eventually dyspnoea. The child used Ventolin when the dyspnoea was severe.

In June 2005, one month before she turned 4, she was treated with Nambudripad's Allergy Elimnation Technique (NAET) [3]. NAET consists of a combination of kinesiology and accupressure. Kinesiology (also called Muscle Response Testing) was used to diagnose the allergies. Subsequently, acupressure (stimulating specific points on the acupuncture meridians) was applied while the child was in contact with the allergen. After each treatment the child had to refrain from contact with the allergen for 24 hours. One allergy was treated at each treatment session. After 7 treatment sessions (within a period of 4 weeks), the intolerable foods were reintroduced one by one. No adverse reactions were observed. After two months, the child was able to eat everything without symptoms. Six months later, she had a relapse after the introduction of new toothpaste, and developed eczema again after milk consumption. She was treated with NAET for titanium dioxide (a whitener, included in toothpastes) and again for milk. After three treatment sessions all adverse reactions were eliminated. After 3 years, in June 2008, she can still eat everything and is free of symptoms.

## Discussion

NAET is a non-invasive, drug-free, holistic treatment, based on a combination of knowledge and techniques borrowing from Western medicine (allopathic) and traditional Oriental medicine (e.g. chiropractic, kinesiology, and acupuncture). According to Chinese medicine, founded thousands of years ago, health is defined as a state of balance within an individual and between the individual and nature. The body is thought to be functioning only in the presence of vital energy, flowing through energy pathways, called meridians. When there is a disruption in the energy flow through the meridians (an increase or decrease) energy blockages can occur, causing symptoms and illness [3-5]. A disruption in the energy flow can be caused by any physical or psychological trauma, such as infections, organ dysfunctions, or emotional stress. Meridian theory explains the mechanisms of energy blockages in the energy meridians and how energy blockages lead to functional imbalances and diseases [4,5].

According to NAET therapists, an allergy is considered to be an energy imbalance between the electromagnetic energy of the person and the allergen. It is hypothesized that measurable weakness in particular muscles is caused by the generation of an energy disturbance in the particular spinal nerve route that supplies to the corresponding weakened muscle when a specific item is brought into its energy field. Muscle Response Testing is based on this hypothesis. Any item that is capable of producing energy disturbance in any spinal nerve route is considered an allergen. No distinction is being made between IgE mediated allergies, cell-mediated allergies, hypersensitivities or intolerances [3].

It is further proposed that by stimulating the specific afferent and efferent nerves and nociceptors in dorsal root ganglia (acupressure) while the person is in contact with the allergen, the NAET treatment is able to change the characteristic of the previous stimulus into a new one with a different signal. These altered stimuli will carry new information about the antigen to the appropriate areas of the cerebral cortex. In return, the brain will relay its response about this new information to every cell in the body. It takes 24 hours to pass the new information through all 12 major meridians. The previously activated immune response will get deactivated or replaced by a new relayed signal. The previously perceived harmful substance is now recognized as being relatively harmless [3].

NAET therapy was developed by a single person, and is considered a controversial therapy by some people. However, NAET is uninvasive, relatively inexpensive and easily administered. Therefore, it might be worthwhile to study the efficacy of NAET in randomized clinical trials, especially in children with food allergy. This has not been done yet.

## Conclusion

This case report highlights the possible benefit of NAET for children with food allergy. Considering the burden of food allergy, especially to young children, randomized clinical trials should be encouraged to study the effectiveness of NAET in treating food allergy.

## Consent

Written informed consent was obtained from the patient's father for publication of this case report. A copy of the written consent is available for review by the Editor-in-Chief of this journal.

## Competing interests

The author is the mother of the case.
